# A single trophectoderm biopsy at blastocyst stage is mathematically unable to determine embryo ploidy accurately enough for clinical use

**DOI:** 10.1186/s12958-017-0251-8

**Published:** 2017-04-27

**Authors:** Norbert Gleicher, Jacob Metzger, Gist Croft, Vitaly A. Kushnir, David F. Albertini, David H Barad

**Affiliations:** 1 The Center for Human Reproduction, 21 East 69th Street, New York, NY 10021 USA; 2 Foundation for Reproductive Medicine, New York, NY 10022 USA; 3 Laboratory of Stem Cell Biology and Molecular Embryology, The Rockefeller University, New York, NY 10065 USA; 4 Department of Obstetrics and Gynecology, University of Vienna School of Medicine, 1090 Vienna, Austria; 5 Center for Studies in Physics and Biology, The Rockefeller University, New York, NY 10065 USA; 6 Department of Obstetrics and Gynecology, Wake Forest University, Winston Salem, NC 27109 USA

**Keywords:** Premplantation genetic screening, Trophectoderm biopsy, Mathematical model, In vitro fertilization

## Abstract

**Background:**

It has become increasingly apparent that the trophectoderm (TE) at blastocyst stage is much more mosaic than has been appreciated. Whether preimplantation genetic screening (PGS), utilizing a single TE biopsy (TEB), can reliably determine embryo ploidy has, therefore, increasingly been questioned in parallel.

**Methods:**

We for that reason here established 2 mathematical models to assess probabilities of false-negative and false-positive results of an on average 6-cell biopsy from an approximately 300-cell TE. This study was a collaborative effort between investigators at The Center for Human Reproduction in New York City and the Center for Studies in Physics and Biology and the Brivanlou Laboratory of Stem Cell Biology and Molecular Embryology, the latter two both at Rockefeller University in New York City.

**Results:**

Both models revealed that even under best case scenario, assuming even distribution of mosaicism in TE (since mosaicism is usually clonal, a highly unlikely scenario), a biopsy of at least 27 TE cells would be required to reach minimal diagnostic predictability from a single TEB.

**Conclusions:**

As currently performed, a single TEB is, therefore, mathematically incapable of reliably determining whether an embryo can be transferred or should be discarded. Since a single TEB, as currently performed, apparently is not representative of the complete TE, this study, thus, raises additional concern about the clinical utilization of PGS.

## Background

It is still widely presumed that elimination of aneuploid embryos prior to embryo transfer improves implantation and clinical pregnancy rates, and reduces spontaneous miscarriages. Preimplantation genetic screening (PGS) is, therefore, clinically still widely utilized [1, 2], though so far has failed to produce expected outcome improvements [3, 4]. Individual studies and meta-analyses, indeed, suggested that the procedure may affect IVF outcomes adversely [5–9]. Challenging the biological concept of PGS, questions also arose whether a single trophectoderm (TE) biopsy (TEB), indeed, can reliably reflect ploidy of the total TE, how accurately a TE biopsy represents the inner cell mass (ICM), from which the embryo arises, and how extensively an embryo self-corrects downstream from blastocyst stage [10, 11].

**Fig. 1:**
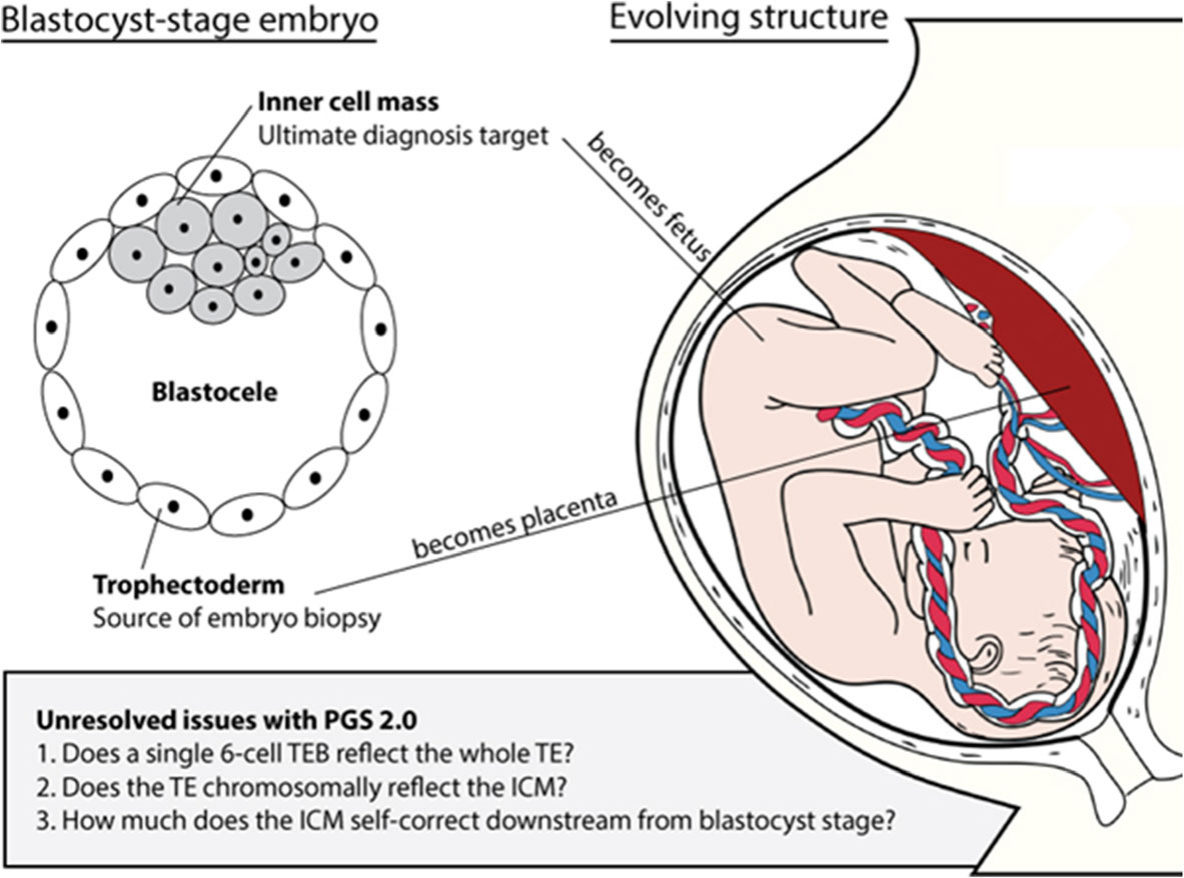
Schematics of PGS. The currently in use PGS procedure (PGS 2.0) is based on a single TEB of on average approximately 6 cells, which is alleged to accurately reflect the chromosomal status of the developing embryo/fetus. The procedure has entered worldwide clinical use without prior clinical validation. Three crucial questions (unresolved issues in the figure) still require resolution. This manuscript attempts to answer the first of these questions, whether a single TEB accurately enough reflects the whole TE to discriminate between embryos that can undergo transfer and those that should be discarded

That embryos self-correct to highly significant degrees was strongly suggested in a recent mouse study, when early stage embryos, even when highly chimeric for euploid and aneuploid cell lineages, remarkably self-corrected downstream from blastocyst stage. Moreover, self-correction was more efficient within the ICM than within TE, from which the placenta develops (Fig. 1) [12]. Faced with such genetic heterogeneity between early embryonic compartments, more aneuploid cells would, therefore, be expected in TE than ICM. Yet, in the current utilization of PGS (PGS 2.0), embryo biopsies are exclusively obtained from the TE.

Until very recently, any detectable aneuploidy in a single TEB was labeled as abnormal (aneuploid), and resulted in disposal of so-affected embryos. Recent studies, however, suggested that such an approach results in large numbers of false-positive diagnoses, exclusion of patients from access to embryo transfers, and disposal of normal (euploid) embryos [10, 11] with potential for normal euploid births [11, 13, 14]. A recent Position Statement on PGS from the *Preimplantation Genetic International Society (PGDIS)*, therefore, defined radically new diagnostic criteria for PGS (Tables 1, 2 and 3) [15].

**Table 1:** *PGDIS* Recommendations for PGS laboratories [15]

1. For reliable detection of mosaicism, ideally 5 cells should be biopsied, with as little cell damage as possible. If the biopsy is facilitated using a laser, the identified contact points should be minimal and preferably at cell junctions. Overly aggressive use of the laser may result in cell damage and partial destruction of cellular DNA.
2. Only a validated Next Generation Sequencing (NGS) platform that can quantitatively measure copy numbers should be used for measurement of mosaicism in the biopsy sample. Ideally, a NGS methodology that can accurately and reproducibly measure 20% mosaicism in a known sample.
3. For reporting embryo results, the suggested cut-off point for definition of mosaicism is >20%, so lower levels should be treated as normal (euploid), > 80% abnormal (aneuploid), and the remaining ones between 20 and 80% mosaic (euploid-aneuploid mosaics).

**Table 2:** *PGDIS* recommendations for the clinician [15]

1. Patients should continue to be advised that any genetic test based on sampling one or small number of cells biopsied from preimplantation embryos cannot be 100% accurate for a combination of technical and biological factors, including chromosome mosaicism.
2. The patient information and consent forms for aneuploidy testing (if used) should be modified to include the possibility of mosaic aneuploid results and any potential risks in the event of transfer and implantation. This needs to be explained to patients by the clinician recommending the aneuploidy testing.
3. Transfer of blastocysts with a normal euploid result should always be prioritized over those with mosaic aneuploid results.
4. In the event of considering the transfer of a blastocyst with only mosaic aneuploidies, the following options should be discussed with the patient:
a. A further cycle of IVF with aneuploidy testing to increase the chance of identifying a normal euploid blastocyst for transfer
b. Transfer of a blastocyst with mosaic aneuploidies for low risk chromosomes only, after appropriate genetic counseling if available
c. Appropriate monitoring and prenatal diagnosis of any resulting pregnancy, preferably by early amniocentesis (14 weeks gestation).

**Table 3:** *PGDIS* guidelines to prioritize mosaic embryos for transfer [15]

Based on our current knowledge of the reproductive outcomes of fetal and placental mosaicism from prenatal diagnosis, the following can be used as a guide by the clinician (or a genetic counselor if available) when a mosaic embryo is being considered for transfer:
1. Embryos showing mosaic euploid/monosomy or mosaic euploid/ monosomy are preferable to euploid/trisomy, given that monosomic embryos (excepting 45, X) are not viable
2. If a decision is made to transfer mosaic embryos trisomic for a single chromosome, one can prioritize selection based on the level of mosaicism and the specific chromosome involved
a. The preferable transfer category consists of mosaic embryos trisomic for chromosomes 1, 3, 4, 5, 6, 8, 9, 10, 11, 12, 17, 19, 20, 22, X, Y. None of these chromosomes involve the adverse characteristics enumerated belowb.
b. Embryos mosaic for trisomies that are associated with potential for uniparental disomy are of lesser priority
c. Embryos mosaic for trisomies that are associated with intrauterine growth retardation (chromosomes 2, 7, 16) are of lesser priority.
d. Embryos mosaic for trisomies capable of liveborn viability (chromosomes 13, 18, 21) are for obvious reasons of lowest priority

The *PGDIS*, thus, acknowledged that prior reporting schemes over-diagnosed aneuploidy. Especially patients with small embryo numbers will be negatively affected by such false-positive diagnoses [2–4, 6]. The society also acknowledged that not only euploid but also selected mosaic embryos now may be transferred, and that a single TEB, therefore, likely is unable to accurately determine whether an embryo is euploid, mosaic-benign or aneuploid.

If a single TEB is unable to offer an accurate diagnosis that allows determination of the ploidy status of the embryo with certainty, the clinical purpose for the utilization of PGS in IVF becomes unclear. This study, therefore, explored with mathematical modeling the most basic question of PGS, −whether a single random TEB can define the expected prevalence of more than one cell clone/lineage in TE of blastocyst-stage embryos.

Since PGS depends on a single TEB, the prevalence of TE mosaicism and/or outright aneuploidy in a single TEB is key to the potential clinical utility of such a biopsy. If frequency of mosaicism makes it impossible for a single biopsy to be representative of total TE, PGS would be invalidated, without even having to consider how well TE reflects the ICM or how much such a biopsy reflects final embryo fate in view of the embryo’s innate ability to self-correct downstream from blastocyst stage.

## Methods and results

### The probability of a false negative diagnosis when biopsy shows no mosaicism

A tempting conclusion from observing only euploid cells in a biopsy is that the whole embryo is euploid. However, there is also the risk of, by chance, not having picked any aneuploid cells in the biopsy, even though some were present. This risk of this happening depends on the number of aneuploid cells in the embryo. We here determine the probability that a biopsy shows no mosaicism as a function of the fraction of euploid cells *r* = ${N}_1/ N$, where ${N}_1$ is the number of euploid cells, and *N* the total number of cells.

**Fig. 2:**
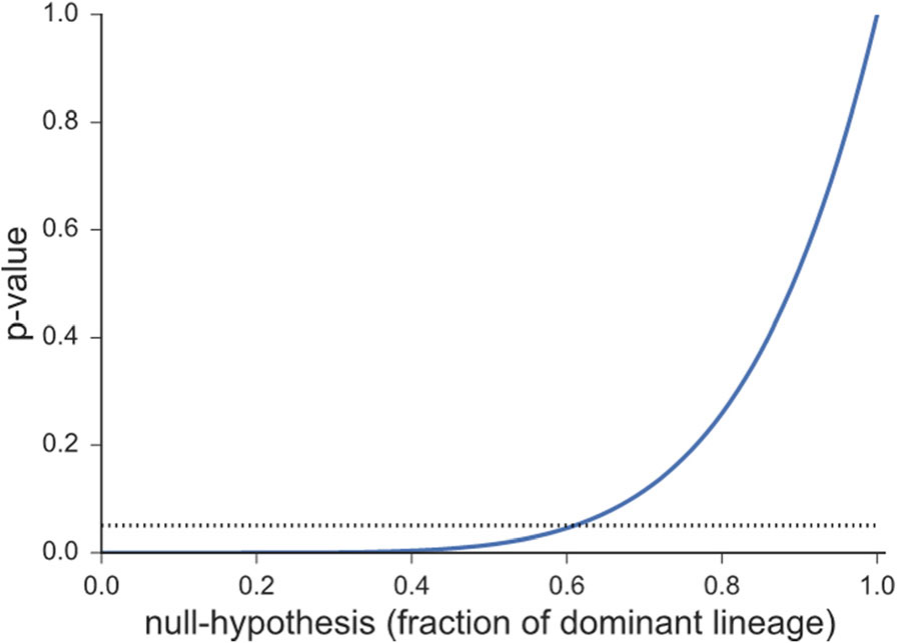
*P*-values for observing no mosaicism, given different hypotheses r and a threshold of 0,05 (*dotted line*). With the curve crossing the significance line (*P* = 0.05) at 0.6, the figure demonstrates that any value of r larger than 60% euploid cells leads to a P-value that is larger than the usual significance level of 5% (indicated as a *dashed line* in the figure), meaning that even the hypothesis that there are 40% aneuploid cells in the embryo cannot be rejected when not observing any aneuploid cells in the biopsy

Assuming that the cells are drawn at random from the trophectoderm, the probability of observing k euploid cells in an n-cell biopsy is given by the hypergeometric distribution $h\left( k, n,{N}_1, N\right)=\left(\genfrac{}{}{0pt}{}{N_1}{k}\right)\left(\genfrac{}{}{0pt}{}{{N- N}_1}{n- k}\right)/\left(\genfrac{}{}{0pt}{}{N}{n}\right)$. The probability of not observing any aneuploid cells in the biopsy although there are some present (i.e. *r* < 1), is given by observing only euploid cells (k = n) which gives

1 $$ p\left( n, n,{N}_1, N\right)=\left(\begin{array}{c}\hfill {N}_1\hfill \\ {}\hfill k\hfill \end{array}\right)/\left(\begin{array}{c}\hfill N\hfill \\ {}\hfill n\hfill \end{array}\right), $$

and which is plotted for different ratios r in Fig. 2 for *n* = 6 (6 cells per TEB) and *N* = 300 (300 cells per average TE at blastocyst stage).

We note that there is a high probability of not observing mosaicism in the biopsy even when the ratio of euploid cells is relatively low. In particular, even a quite mosaic embryo with only 60% euploid cells leads to observing no mosaicism in the biopsy in more than 5% of the cases. In more technical terms, equation (1) is the *P*-value for the hypothesis that the ratio of euploid cells is r. It shows that any value of r larger than 60% euploid cells leads to a *P*-value that is larger than the usual significance level of 5% (indicated as a dashed line in the figure), meaning that even the hypothesis that there are 40% aneuploid cells in the embryo cannot be rejected when not observing any aneuploid cells in the biopsy.

One can also ask the reverse question; i.e. how large would the biopsy need to be so that one can be confident that the embryo consists of at least a certain high fraction of euploid cells. We can calculate the size of the biopsy for which it would very likely (>95%) not be all-euploid, by equating expression (1) with 0.05 and by then solving for the biopsy size n. For example, one would need a biopsy of 27 cells to assure that the probability of obtaining only euploid cells in a biopsy is less than 5% if the fraction of euploid cells in the embryo is *r* = 0.9 (i.e. for a biopsy size of *n* = 27, only hypotheses *r* > 0.9 are compatible with an all-euploid biopsy, and in 95% of the cases one would observe a mosaic biopsy).

### The probability of false positive diagnosis when biopsy shows mosaicism

We now calculate the probability that the embryo is, to a significant degree, normal-euploid and non-mosaic (i.e., can have a large r), even though the biopsy includes one or more aneuploid cells. Similar to the above calculation, we are interested in the largest r that is still compatible with a biopsy that includes aneuploid cells. We analyze the case of different numbers of aneuploid cells in the biopsy separately. The most extreme (i.e. unlikely case, assuming that there is a dominating constitutional lineage) is that all 6 cells are abnormal-aneuploid, which is given by the hypergeometric distribution with k = 0 (no euploid cells), leading to

2 $$ p\left(0, n,{N}_1, N\right)=\left(\begin{array}{c}\hfill N-{N}_1\hfill \\ {}\hfill n\hfill \end{array}\right)/\left(\begin{array}{c}\hfill N\hfill \\ {}\hfill n\hfill \end{array}\right) $$

This gives us the *P*-value or probability that, for a given ratio of euploid cells in the embryo, r, 6 aneuploid cells are observed. If there are 5 abnormal-aneuploid cells, the *P*-value is calculated by adding up the probability of getting 5 abnormal cells and the more extreme case of 6 abnormal cells, and so on for the other cases, leading to a *P*-value ${p}_i$ (indicating a biopsy with *i* abnormal cells) of

3 $$ {p}_i={\displaystyle {\sum}_{j= i}^n h\left( n- j, n,{N}_1, N\right)} $$

**Fig. 3:**
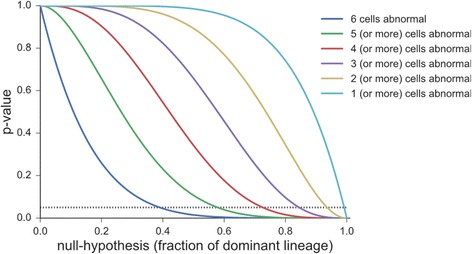
*P*-values for observed mosaicism, given different hypotheses r, and varying numbers of abnormal-aneuploid cells in biopsy. The curves demonstrate that, even when obtaining a mosaic TEB, with decreasing aneuploidy cell numbers in the TEB (from 6 to 1), any explanation with increasing r from *r* < 0.4 to *r* < 0.99 could be a reasonable explanation of the observed data since it leads to observing mosaicism in >5% of cases. The threshold at which r crosses the significance level increases with decreasing aneuploidy. This means that a given r is more likely to explain the obtained biopsy result the lower the measured aneuploidy is. For example, if one (or more) cells are aneuploid, the threshold will be approximately *r* = 0.99, meaning that even a hypothesis of *r* = 0.99, basically an entirely euploid embryo, is compatible with the data. On the other hand, if three (or more) cells are aneuploid, a hypothesis with an r as high 0.99 is too unlikely to give the observed data, and only an *r* < 0.85 hypothesis is compatible with this biopsy outcome

The *P*-values for different biopsies outcomes and hypotheses is, therefore, plotted in Fig. 3. The figure allows easy determination of the largest r that cannot be rejected (i.e. where the lines cross the significance level).

As already explained above, in determining the interpretation of a TEB negative for aneuploid cells, the curves in Fig. 3 demonstrate that, even when obtaining a mosaic TEB, with decreasing aneuploidy cell numbers in the TEB (from 6 to 1), any explanation with increasing r from *r* < 0.4 to *r* < 0.99 could be a reasonable explanation of the observed data since it leads to observing mosaicism in >5% of cases. The threshold at which r crosses the significance level increases with decreasing aneuploidy. This means that a given r is more likely to explain the obtained biopsy result the lower the measured aneuploidy is. For example, if one (or more) cells are aneuploid, the threshold will be approximately *r* = 0.99, meaning that even a hypothesis of *r* = 0.99, basically an entirely euploid embryo, is compatible with the data. On the other hand, if three (or more) cells are aneuploid, a hypothesis with an r as high 0.99 is too unlikely to give the observed data, and only an *r* < 0.85 hypothesis is compatible with this biopsy outcome.

What models for false-negative and false-positive diagnoses, therefore, demonstrate is that, in both instances, the uncertainty about accuracy is so high that a single TEB offers limited clinical value for determining prevalence and degree of TE aneuploidy. And this uncertainty does not even yet relate to the uncertainty about how TE aneuploidy relates to ICM aneuploidy and, finally, to the apparently remarkable ability of the ICM to self-correct, even if at blastocyst stage still highly chimeric between a constitutional euploid and non-constitutional aneuploidy cells (Fig. 1 and [12]).

Two additional points are of importance: Here presented models are best case scenarios because they assume an even distribution of non-constitutional cell lines throughout the TE, which in itself is unlikely. More likely is a clonal insular distribution akin to aneuploid cell island described in the placenta [16], which arises from the TE. Such a distribution would, however, further lower here already inadequately low predictive abilities of a single TEB. In addition, both here presented models clearly demonstrate that even the new *PGDIS* criteria to define embryos as normal, mosaic and aneuploid based on percentages of aneuploidy (Table 1), are unsustainable because they do not permit the one decision why PGS 2.0 is performed by clinicians, − to determine whether embryos can be transferred or should be discarded. As our second model (Fig. 3) demonstrates, even 80% aneuploidy in a single TEB is compatible with the hypothesis of an embryo being approximately 60% euploid. Even a level of 80% aneuploidy in a single TEB, therefore, cannot be used as a convincing rational to discard so assessed embryos. One, therefore, has to wonder what the remaining clinical purpose of PGS 2.0 might be?

## Discussion

Embryos with any chromosomal abnormality were until recently routinely discarded before publication of the new *PGDIS* criteria [15]. Figure 3 demonstrates that, based on the ratio of euploid/aneuploidy cells in a TEB, different degrees of mosaicism can be considered reasonable explanations of the data, with declining numbers aneuploid cells in the TEB decreasing the likelihood that a given ratio of euploid/aneuploid cells can explain the data observed.

Decreasing aneuploidy increases the *P*-value for a given r, and also increases the threshold r, for which the *r* < r threshold is a valid explanation for the biopsy data. Since a very high r means almost no mosaicism, decreasing numbers of aneuploid cells in the TEB lead to the hypothesis that an increasingly non-mosaic euploid embryo has to be considered a reasonable explanation for the observed biopsy results.

Here presented models assume that a single TEB on average involves 6 TE cells, and that a healthy blastocyst stage embryo demonstrates a TE of approximately 300 TE cells. The latter number is based on a recently published human *in vitro* implantation model [17] and differs from TE cell numbers reported in earlier studies obtained in embryos from preimplantation-stage blastocysts, which suggested a smaller TE cell pool of ca.70-120 cells [18, 19].

Even assuming the TE cell pool to be smaller than 300 cells, magnitude of here reported mathematical uncertainty beyond reasonable doubt demonstrates that a single TEB cannot reflect the total TE. Even under the unrealistic assumption of even distribution of mosaicism, it would take at least a 27-cell biopsy to reach a minimal level of correct statistical representation. A 6-cell biopsy is, therefore, never accurate enough to decide whether an embryo should be discarded or not.

Here presented mathematical models also offer a likely explanation why reported live birth rates after transfer of allegedly aneuploid/mosaic embryos have been surprisingly high [11, 13, 14], and why these results contradict newly published *PGDIS* criteria, attempting to redefine clinically relevant degrees of embryo mosaicism (i.e., “euploid-normal” <20% aneuploidy, “euploid-aneuploid mosaic” at ≥20–80%, and “abnormal-aneuploid” at >80%) ([15] and Table 1). The *PGDIS* also defines a TEB by only 5-cells, thereby further reducing the potential predictability of a single biopsy, and more than compensating should the assumption of 300 TE cells in our models have been too high [18, 19]. A 5-cell TEB also means that 1 to 4 aneuploid cells in a single TEB define an embryo as mosaic. As this new *PGDIS* guideline also sets <20% aneuploidy for the definition of euploid-normal (Table 1), an embryo under this new definition, therefore, would be “normal” only if a single TEB contains zero aneuploid cells.

The *PGDIS’* new definitions of euploid-normal, mosaic and aneuploidy-abnormal, therefore, defy logic on theoretical as well as practical grounds. They also offer a good example why genetic test validations should not be based on technical limits of diagnostic platforms but on appropriate clinical validation studies before such platforms are introduced into routine clinical practice.

How mosaic TE is, how many individual cell clones are spread throughout the TE, how expansive each clone is and how individual clones are distributed among TE cells (sticky-clustered or evenly distributed) is, of course, essential to any understanding of how well (or poorly) a single TEB represents the whole TE. Not one of these defining factors of mosaicism is, however, currently known.

Munné’s group recently demonstrated how limited current knowledge is [20]: Utilizing array comparative genomic hybridization (CGH) and next generation sequencing (NGS), they analyzed the age-prevalence of chromosomal abnormalities. Among large numbers of investigated embryos, CGH (which cannot differentiate between aneuploid and mosaic embryos) at all ages defined more embryos as aneuploid than NGS, which is claimed to differentiate between euploid, mosaic and aneuploid embryos based on percentages of aneuploidy ([15] and Table 1).

Adding, however, up aneuploid and mosaic findings after NGS, we noted that NGS at all ages detected significantly more non-euploid embryos than CGH. Though aneuploid embryos increased with advancing age with both platforms, the study’s authors noted that mosaicism in infertile women, actually, significantly decreased with advancing age. Incompatible with this finding, and unexplained by the authors, egg donors, however, paradoxically demonstrates almost as low mosaicism rates as oldest women (>40 years) [20].

How a single random TEB, involving ca. 6 cells [15, 21] in a ca. 300 cell T [17], reflects the overall chromosomal heterogeneity of the total TE, was, until here reported mathematical models were applied, unknown. PGS 2.0 was, nevertheless, clinically initiated without proper prior validation studies. This is especially noteworthy since a main argument for advancing from PGS 1.0 (utilizing blastomere biopsy at cleavage stage) to PGS 2.0 (TEB at blastocyst stage) was allegedly reduced mosaicism [22].

Recently evolving data suggest the opposite: Like cancer cells, blastomeres of early stage human embryos exhibit increased expression of gene products involved in cell cycle progression, while apparently lacking expression of cell cycle checkpoint genes. Convergence of these two properties at blastocyst stage, due to increased mitotic error rates [23], may at least in part explain increased genetic instability and increasing TE mosaicism. Stress imposed by extended embryo culture may also contribute to increased mosaicism [24]. PGS 1.0 years ago was declared ineffective in improving IVF outcomes [25–28]. As here presented models suggest, PGS 2.0 appears on the way to meet a similar fate,.

Reports on the prevalence of TE mosaicism at blastocyst stage have not been consistent: An Italian group reported between 3 to 8% [14, 29]. As noted, Munné’s group reported that TE mosaicism declined with advancing age (from 22.66% <35 years to 9.70% >42). Paradoxically, young egg donors, however, behaved like oldest women by demonstrating mosaicism in only 11.35% [20]. Adding up euploid and mosaic embryos, percentages, however, suddenly make age-specific sense, with young egg donors demonstrating lowest aneuploidy/mosaicism rates (26.06%), gradually increasing to 79.54% in oldest infertile women above age 42.

Most so far published PGS studies in the literature have not defined mosaicism in accordance with recently issued *PGDIS* guidelines, nor did they use, as suggested, NGS platforms (Table 1). They, therefore, can no longer be considered authoritative. The new *PGDIS* criteria, however, are also still insufficient since they are based on alleged sensitivities of selected NGS platforms to detect mosaicism quantitatively. Yet, they lack any clinical validation studies.

For the third time in the history of PGS, the PGS laboratory community, therefore, without prior clinical validation studies, has chosen to establish completely arbitrary diagnostic criteria to differentiate between euploid (<20% aneuploidy), mosaic (20–80% aneuploidy) and aneuploid embryos (>80% aneuploidy [15], claiming that such differentiation can be the basis for clinical decisions on whether to transfer or dispose of any given embryo. We here demonstrate the futility of this claim.

TE mosaicism significantly exceeds previously reported rates: Less than 20% of embryos were identically assessed on multiple biopsies in different PGS laboratories [11]. Over a-third of embryos initially reported as aneuploid, on repeat biopsy were found to be euploid-normal [11]. Evaluating multiple TEB biopsies from same embryos in same PGS laboratories, only approximately 50% of biopsies were congruent [11]. TE mosaicism, mosaicism, therefore, exists in, likely, at least 50% of embryos and with considerable certainty in close to 100%. Congruence between multiple TEBs and ICM biopsies was also poor [10], offering additional evidence that a single TEB cannot reliably determine the chromosomal status of a blastocyst stage embryo. As noted before, based on increasing chromosomal instability with extended embryo culture [23, 24], TE mosaicism may actually reflect a normal physiological stage in embryo development in preparation for implantation. Munné’s group’s suggestion that mosaicism declines with advancing maternal age [20], could then, at least partially, explain declining implantation rates in older women.

## Conclusions

The primary goal of this communication was defining the likelihood of TE mosaicism in human blastocyst-stage embryos. The primary question of interest was, however, how accurately can a single TEB reflect the ultimate chromosomal fate of an embryo? The answer depends on five distinct components: (i) How accurate is the diagnostic platform in determining whether a specimen is euploid, mosaic or aneuploid? (ii) How accurately does this one TEB, involving approximately 6 cells, reflect the total TE? (iii) How well does this one TEB reflect ploidy of the ICM? (iv) What is the age of the mother? (v) How well does the ICM, ultimately self-correct downstream from blastocyst stage?

Results of embryo biopsies can significantly vary between diagnostic platforms [30]. The recent *PGDIS* statement extensively refers to the use of appropriate platforms ([15] and Table 1). Yet, surprisingly, none has so far been vetted by the U.S. Food and Drug Administration (FDA), and even formal comparisons between marketed systems are lacking.

We here confirmed that TE mosaicism must be more common at blastocyst stage than has been initially suggested by proponents of PGS 2.0 [22]. Though our models cannot offer specific probabilities, here presented data strongly suggest that a single TEB cannot accurately enough determine whether, and to what degree, a given embryo is mosaic and/or aneuploid. A single TEB, therefore, is not sufficient to determine whether an embryo can be transferred or should be discarded, rendering PGS 2.0 as a clinical tool ineffective.

How this variability of outcome with a single TEB relates to the ICM is unknown. After dividing embryos into four specimens, Munné’s group recently reported that mosaicism was confined to only TE or ICM in 39% of embryos. In 25% of embryos, the ICM was mosaic, yet, one or two TE biopsies were euploid [29]. Their study, thus, confirms relative poor correlations between TE and ICM, a findings first suggested by Orvieto et al. [10]. This observation, of course, further reduces the relevance of a single TEB in defining embryo ploidy.

Aneuploid cell lineages increase with advancing female age [20], likely increasing the ratio of non-constitutional to constitutional cells and, thereby, further reducing the accuracy of a single TE biopsy. When in older women, accurate diagnosis of ploidy is needed most, PGS 2.0, therefore, appears least accurate. Considering that embryo numbers decline with advancing age, it, therefore, should not surprise that PGS, even in its earlier format (PGS 1.0) already was demonstrated to adversely affect IVF outcomes in older women and poorer prognosis patients [6, 8].

The ultimate question to be answered is, however, how well the ICM, from which the fetus arises, self-corrects downstream from blastocyst stage? If human embryos have a similar ability of self-correction as mice [12], a TEB at blastocyst stage would seem non-sensical. Even, assuming the unlikely ability to biopsy the ICM rather than the TE of blastocyst-stage embryos, results would mean little since the evolving embryo would still have significant capacity to self-correct downstream.

We, therefore, conclude that the basic biology of early embryonic development invalidates the concept of PGS. It, therefore, should not surprise that PGS 1.0 failed and, ultimately, was declared ineffective [24–27], and that increasing clinical data now also suggest ineffectiveness of PGS 2.0.

Recently published national U.S. data from the Centers for Disease Control and Prevention (CDC), comparing outcomes in IVF cycles with and without PGS, suggested potential negative effects from PGS [31]. Analyses of national Society for Assisted Reproduction (SART) data, further strengthened those conclusions by also demonstrating negative outcome effects from PGS, even in best prognosis patients (Barad DH, Darmon S, Kushnir VA, Lazzaroni-Tealdi E, Wang Q, Zhang L, Albertini DF, Gleicher N. Detrimental effects of preimplantation genetic screening (PGS) on 2005-2013 oocyte recipient cycles, In revisions). That prospective studies of PGS were unable to demonstrate outcome benefits from PGS, whether in its first (PGS 1.0) or its current format (PGS. 2.0) [3, 4], should, therefore, not surprise.

Increasing numbers of healthy offspring delivered following transfers of allegedly aneuploid/mosaic embryos [11, 13, 14] have called further into doubt the longstanding policy of discarding such embryos. This was recognized by the *PGDIS* ([15] and Tables 2 and 3), the first formal acknowledgement that, until recently, large numbers of embryos with euploid live birth potential have been mistakenly disposed all around the world.

Some investigators suggested that transfer of mosaic embryos reduces implantation rates in comparison to transfer of euploid embryos [15, 32]. Their conclusions have, however, be viewed with caution since studies that have made this claim utilized diagnostic platforms incapable of accurately discriminating between benign embryo mosaicism and true embryo aneuploidy. There is, indeed, considerable evidence to the contrary: That includes rather high live birth rates from transferred aneuploid/mosaic embryos in mostly poor prognosis patients [11, 13, 14].

To summarize, our mathematical modeling of a single TEB to accurately assess presence of mosaicism, demonstrates that such a single biopsy cannot reliably evaluate ploidy of TE and/or embryo to determine whether an embryo should be transferred or discarded. PGS 2.0, therefore, remains a procedure in search of a clinical application [4], and should not be offered clinically in attempts to improve IVF outcomes.

Finally acknowledging shortcomings of PGS 2.0, the recent Position Statement of the *PGDIS* [15] still falls short, as it does not offer the option of reducing the use of PGS in association with routine IVF. Remarkably, indeed, as first and preferred option in cases where PGS 2.0 only reports mosaic blastocysts in an IVF cycle, the new recommendation is *“a further cycle of IVF with aneuploidy testing to increase the chance of identifying a normal-euploid blastocyst for transfer”* (Table 2). Our study establishes that such a recommendation does not rely on any scientific evidence.

Joyner et al. recently discussed in JAMA the unfortunate tendency of underperforming big ideas in research, nevertheless, to become entrenched in clinical medicine [33]. In reproductive medicine, PGS increasingly looks like the posterchild for an underperforming idea.
